# Changes in Growth and Polyphenol Content of the Rare Plant *Persicaria chinensis* Cultivated in a Greenhouse During the Growth Period

**DOI:** 10.3390/plants15030498

**Published:** 2026-02-05

**Authors:** Daeho Choi, Yong-Woo Park, Jungmok Kang, Hwayong Lee

**Affiliations:** 1 Forest Bio Center, Chungcheongbuk-do Forest Environment Research Center, Okcheon 29061, Republic of Korea; eoghchoi@korea.kr (D.C.); pywcan@korea.kr (Y.-W.P.); kjm1111@korea.kr (J.K.); 2 Department of Forest Science, Chungbuk National University, Cheongju 28644, Republic of Korea

**Keywords:** rare plant, corilagin, ellagic acid, geraniin, neochlorogenic acid

## Abstract

The growth characteristics and changes in major polyphenol content of the rare plant *Persicaria chinensis* cultivated under greenhouse conditions were investigated to evaluate its potential for large-scale cultivation and industrial use. The fresh and dry weights of the leaves and stems were measured monthly from May to October, and the corilagin, ellagic acid, geraniin, and neochlorogenic acid contents were analyzed. Leaf fresh and dry weights peaked in June (11.73 ± 4.74 g and 3.02 ± 1.22 g, respectively) and increased again in August thereafter, and subsequently decreased, whereas stem fresh and dry weights continuously increased throughout the cultivation period, reaching 20.06 ± 3.88 g and 7.68 ± 1.55 g, respectively, in October. The polyphenol content in leaves was generally highest in June and then declined. In September, the contents of corilagin and ellagic acid showed marked increases, reaching 10.34 ± 4.13 mg/g and 7.26 ± 3.78 mg/g, respectively. In the stems, the polyphenol content was lower than that in the leaves and showed a decreasing trend after the early cultivation stage. Correlation analysis revealed weak relationships between biomass and polyphenol content in the leaves, whereas strong positive correlations among polyphenols and negative correlations between stem growth and polyphenol content were observed in the stems. These results demonstrate that stable greenhouse cultivation of *P. chinensis* and the accumulation of functional compounds are feasible and provide fundamental information for the development of cultivation strategies, including appropriate fertilization and environmental management, aimed at functional raw material production.

## 1. Introduction

The perennial herb *Persicaria chinensis* (L.) H. Gross (Polygonaceae) is distributed across East Asia, India, and North America. In Korea, it is a rare plant species restricted to limited coastal areas of Jeju Island and is classified as vulnerable by the International Union for Conservation of Nature (IUCN) [1]. *P. chinensis* has been used in medicinal raw material for the treatment of sore throat and fever [2,3], and exhibits antibacterial, antimicrobial, and anti-inflammatory activities, indicating its potential for use as an industrial material in pharmaceutical, cosmetic, and functional food applications [4,5]. Recent studies of *P. chinensis* have focused on phytochemical screening, antioxidant analyses, and pharmacological evaluations [6,7]. In addition, *P. chinensis* has recently attracted increasing attention as a potential raw material for functional foods and health-related products, and its industrial utilization is currently being explored [8]. In this context, polyphenolic compounds are widely recognized as key quality indicators for the quality control and standardization of functional plant materials [9]. It has been reported that *P. chinensis* native to Korea predominantly contains four polyphenolic compounds with industrial potential: corilagin, ellagic acid, geraniin, and neochlorogenic acid [10]. Corilagin and geraniin have been reported to exhibit various pharmacological activities, including hepatoprotective and anticancer effects [11,12], and ellagic acid has demonstrated anti-inflammatory and neuroprotective properties [13]. Neochlorogenic acid has also been reported to be effective in cancer prevention and treatment [14]. Given their diverse biological activities, these polyphenols can be considered key quality indicators for the industrial evaluation of *P. chinensis*.

As average temperatures continue to rise due to climate change, plant species previously confined to southern Korea are increasingly cultivable in central regions [15,16]. Under the current climate, greenhouse cultivation is necessary to enhance cultivation efficiency and provide a stable growing environment. Greenhouse cultivation, which enables precise control of growth conditions, has been recognized as an effective strategy for producing raw plant materials of uniform quality [17,18]. Greenhouse conditions, which minimize the influence of external weather fluctuations, enabling stable production and consistent levels of bioactive compounds, represent a key cultivation strategy for the quality stabilization of medicinal and functional plants in which the content of bioactive compounds is highly sensitive to environmental conditions [19,20].

Against this background, the development and utilization of rare plant species as novel industrial resources necessitates evaluation of growth and quality characteristics according to cultivation period, as well as studies aimed at enhancing productivity through fertilization and environmental control [21]. The industrial use of rare plants such as *P. chinensis* not only creates added economic value but also offers advantages in terms of resource conservation through the expansion of cultivation areas [22]. However, to date, no studies have reported greenhouse cultivation experiments or analyses of functional compounds in *P. chinensis*. Therefore, this study aimed to investigate fundamental cultivation traits and changes in major polyphenol contents of *P. chinensis* under greenhouse conditions, providing a scientific basis for the development of cultivation strategies and industrial application as a functional raw material.

## 2. Results and Discussion

### 2.1. Environmental Conditions During the Cultivation Period

**Figure 1:**
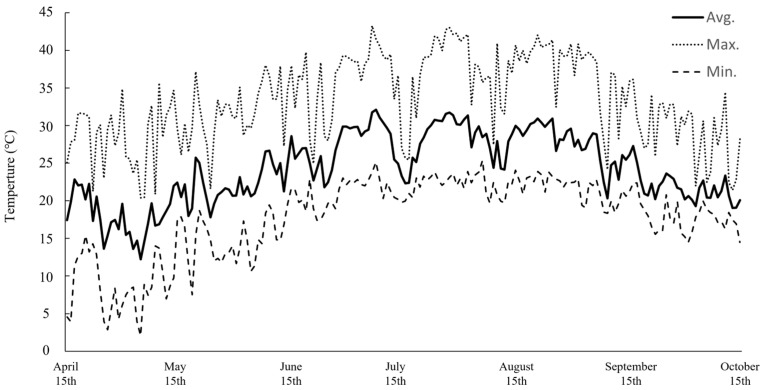
Temperature data of the greenhouse during the cultivation period of *P. chinensis*.

The changes in greenhouse temperature during the experimental period are shown in Figure 1. The average temperature throughout the cultivation period was approximately 20 °C, with the highest mean temperature reaching 25 °C in July and August, after which greenhouse declined. Minimum temperatures during the cultivation period occurred throughout most of April and May, when greenhouse temperatures frequently dropped below 10 °C. Conversely, maximum temperatures exceeding 40 °C occur at times in July and August. Notably, the monthly average temperatures of Seogwipo, Jeju Island, the native habitat of *P. chinensis*, in 2025 were generally comparable to those observed under greenhouse conditions; however, the maximum temperatures from July to September did not exceed 35 °C [23]. This difference suggests that applying temperature management based on native habitat conditions, particularly by mitigating extreme summer heat in the greenhouse, may further enhance plant growth and productivity.

**Figure 2:**
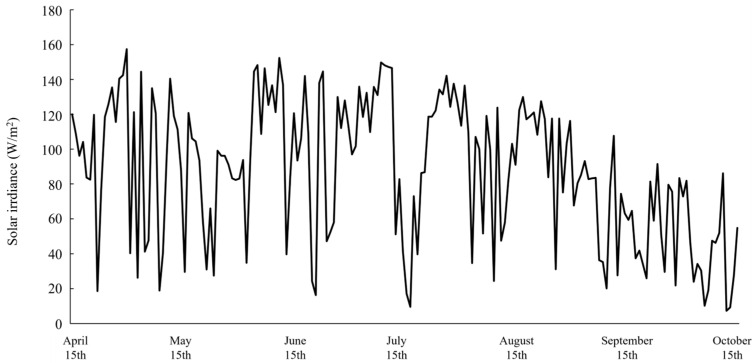
Average solar irradiance data of the greenhouse during the cultivation period of *P. chinensis*.

The changes in the daily average solar irradiance during the experimental period are shown in Figure 2. From mid-April to late August, solar irradiance generally remained at approximately 100 W/m^2^. After August, the solar irradiance decreased to below 80 W/m^2^.

### 2.2. Plant Growth, Fresh Weight, and Dry Weight

**Figure 3:**
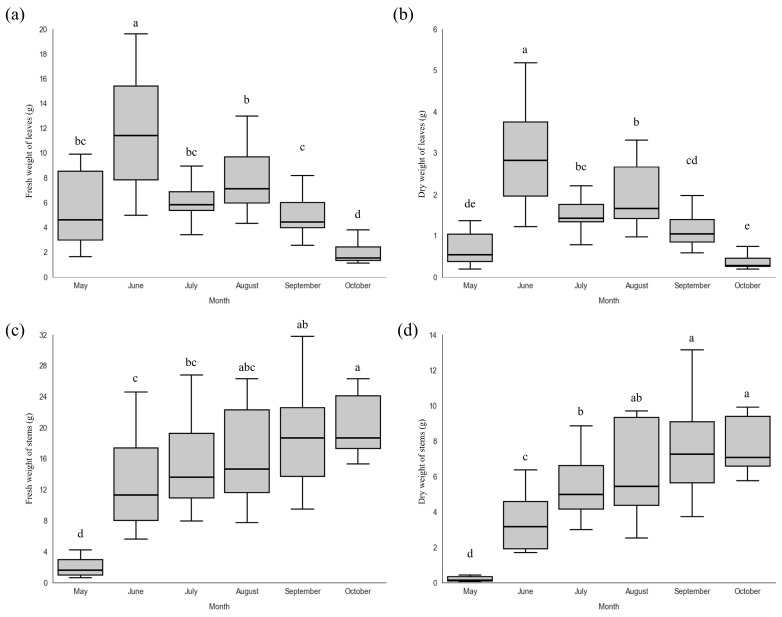
(**a**) Fresh weight of leaves, (**b**) dry weight of leaves, (**c**) fresh weight of stems, and (**d**) dry weight of stems of *P. chinensis* grown in the greenhouse across the cultivation period. Different letters above the columns indicate a significant difference (*p* < 0.05).

To evaluate the utilization potential of *P. chinensis*, plants were cultivated under greenhouse conditions, and the fresh and dry weights of the leaves and stems were measured on a per plant basis at monthly intervals. Leaf fresh weight increased from 6.04 ± 4.33 g in May to 11.73 ± 4.74 g in June, decreased to 6.18 ± 1.56 g in July, and then increased again to 7.99 ± 2.90 g in August (Figure 3a). A similar trend was observed for leaf dry weight, which increased to 3.02 ± 1.22 g in June and subsequently decreased to 1.52 ± 0.40 g in July and 1.98 ± 0.80 g in August (Figure 3b). In contrast, stem fresh weight continuously increased throughout the cultivation period, reaching 20.06 ± 3.88 g per plant in October, and stem dry weight also increased steadily to 7.68 ± 1.55 g per plant by October (Figure 3c,d). Because *P. chinensis* is a rare plant species, previous studies on its growth characteristics in natural habitats are limited. Therefore, future studies should establish cultivation strategies by comparing growth patterns between greenhouse conditions and native habitats.

**Figure 4:**
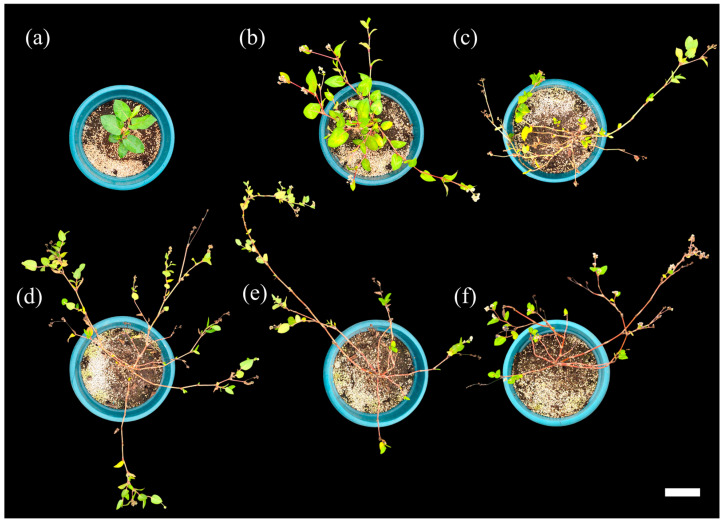
*P. chinensis* grown in the greenhouse across the cultivation period. (**a**) 15 May, (**b**) 15 June, (**c**) 15 July, (**d**) 15 August, (**e**) 15 September, and (**f**) 15 October. Scale bar = 10 cm.

After August, although new leaves continued to form during stem growth, leaf abscission exceeded leaf formation, resulting in most leaves being retained only on the apical parts of the stems. Consequently, leaf fresh and dry weights markedly decreased in October to 1.93 ± 0.75 g and 0.36 ± 0.15 g, respectively. This reduction is likely attributable to decreased solar irradiance after August, which may have led to reduced photosynthetic activity and carbon assimilation, thereby promoting leaf abscission and, consequently, reduced energy consumption [24]. In addition, the decline in temperature after August may have contributed to leaf abscission through decreased auxin levels and increased ethylene production in the plants [25]. Because stem growth continued throughout the cultivation period, these results suggest that preventing leaf abscission and promoting sustained vegetative growth could potentially enhance overall biomass production (Figure 4).

**Figure 5:**
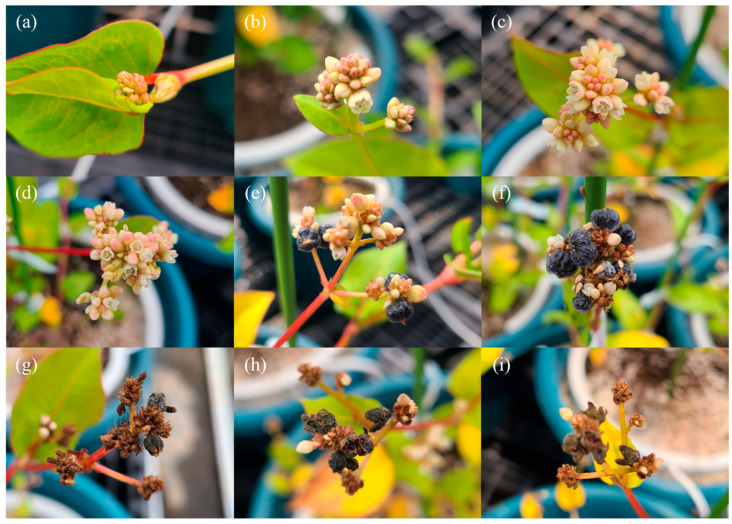
Reproductive development of *P. chinensis* at different time points: (**a**) May 29th, (**b**) 4 June, (**c**) 10 June, (**d**) 15 June, (**e**) 23 June, (**f**) 26 June, (**g**) 30 June, (**h**) 4 July, and (**i**) 10 July.

The reproductive growth of *P. chinensis* begins in late May, when flower buds start to form at the shoot apices of most individuals. In the greenhouse plants, full flowering occurred by mid-June, followed by fruit set, and the entire reproductive phase occurred over a period of approximately 40 days (Figure 5). Fruit maturation from late June onward may limit the supply of nutrients required by the leaves, thereby contributing to leaf abscission. After completion of the initial flower clustering in July, clusters of flowers continued to form at the apical ends of actively growing shoots in individual plants. This pattern is consistent with previous reports by Elliott and Starr [26], indicating the continuous flowering of *P. chinensis* throughout the growing season. These results suggest that *P. chinensis* exhibits an indeterminate flowering type, which facilitates the continuous production of flowers and fruits, and implies that floral and fruit tissues may also be considered as potential harvestable plant parts [27]. In indeterminate flowering plants, nitrogen-oriented fertilization during the early vegetative stage and balanced fertilization during the continuous flowering stage, when vegetative and reproductive growth occur simultaneously, are considered effective management strategies [28,29,30], Further studies are required to establish optimal fertilization regimes for *P. chinensis* based on these growth characteristics.

### 2.3. Corilagin, Ellagic Acid, Geraniin, and Neochlorogenic Acid Content

**Figure 6:**
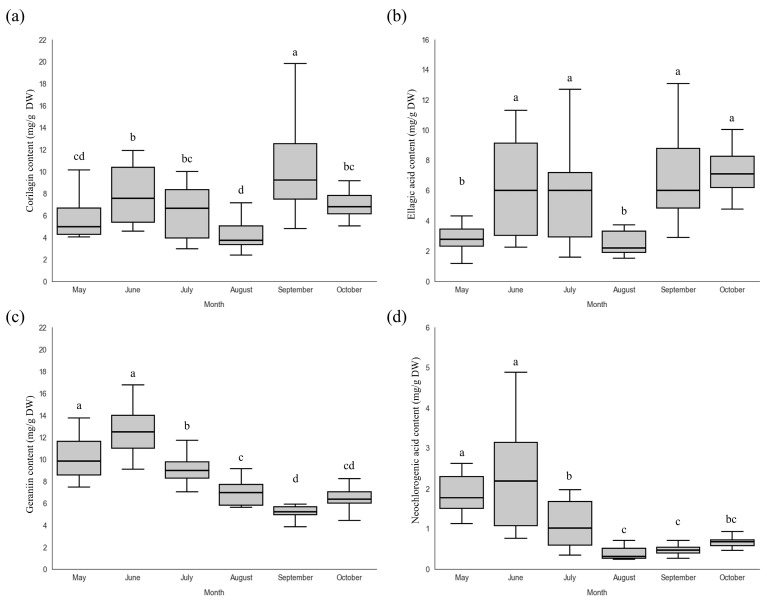
(**a**) Corilagin, (**b**) ellagic acid, (**c**) geraniin, and (**d**) neochlorogenic acid contents in leaves of *P. chinensis* grown in the greenhouse across the cultivation periods. Different letters above the columns indicate a significant difference (*p* < 0.05).

All polyphenol contents are expressed on a dry weight basis (mg/g DW). The corilagin content in the leaves of *P. chinensis* increased to 7.89 ± 2.73 mg/g by June, then decreased until August, and subsequently increased again to 10.34 ± 4.13 mg/g in September. Throughout most of the cultivation period, the corilagin content remained above 4 mg/g. Ellagic acid content increased to 6.16 ± 3.21 mg/g by June, decreased until August, and then increased again to 7.26 ± 3.78 mg/g in September, showing a trend similar to that of corilagin. Ellagic acid levels were maintained above 2 mg/g during the cultivation period and exhibited greater variability than did the other polyphenols. Geraniin showed the highest content among the analyzed polyphenols, reaching a maximum of 12.59 ± 2.21 mg/g in June, followed by a decreasing trend and a slight increase to 6.37 ± 1.16 mg/g in October. The geraniin content remained above 5 mg/g throughout the cultivation period. Neochlorogenic acid exhibited a trend similar to that of geraniin, increasing to 2.22 ± 1.32 mg/g in June, decreasing thereafter, and slightly increasing again to 0.68 mg/g in October; however, it consistently showed the lowest content among the polyphenols detected in *P. chinensis* leaves (Figure 6). To date, no studies have systematically investigated seasonal changes in the contents of the four polyphenols analyzed in this study within the genus *Persicaria* or the family Polygonaceae, which limits direct comparisons with the same or closely related species. Therefore, the present results were interpreted by referring to previous studies on other plant species containing similar secondary metabolites. Previous studies have reported that ellagic acid content in leaves of *Punica granatum* (Lythraceae) continuously increased from June to October [31], geraniin content in leaves of *Geranium sylvaticum* (Geraniaceae) was highest after the seed maturation stage in July, and neochlorogenic acid content in leaves of *Eriobotrya japonica* (Rosaceae) exhibited minimal seasonal variation [32,33]. These differences are likely attributable to the species-specific life cycles and physiological characteristics. In the present study, the overall polyphenol content in *P. chinensis* leaves was highest in June, coinciding with the period of the most active leaf growth and metabolic activity. As leaf abscission progressed, a general decrease in polyphenol content was observed, and polyphenol levels were further reduced in association with physiological changes, such as fruit maturation [34]. Therefore, the continued development of cultivation techniques and further investigation of polyphenol dynamics are required to support the effective utilization of *P. chinensis*.

**Figure 7:**
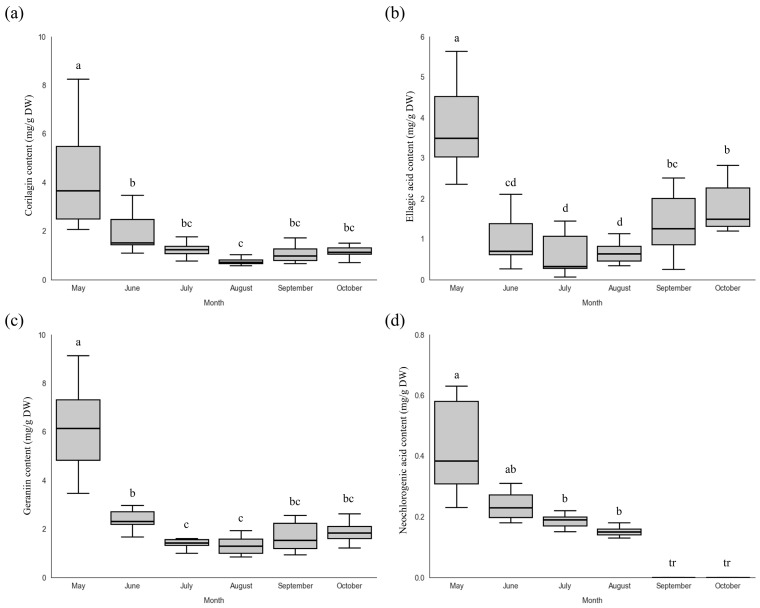
(**a**) Corilagin, (**b**) ellagic acid, (**c**) geraniin, and (**d**) neochlorogenic acid contents in stems of *P. chinensis* grown in the greenhouse across the cultivation periods. Different letters above the columns indicate a significant difference (*p* < 0.05). tr: trace level.

The concentration of the four polyphenols in the stems of *P. chinensis* was generally lower than what was found in the leaves (Figure 7). This result is consistent with previous reports indicating that polyphenol content tends to be higher in leaves than in stems [35], as is also the case in *P. chinensis* in particular [10]. Corilagin content was highest at the early cultivation stage in May (4.52 ± 2.64 mg/g), followed by a marked decrease and a slight increase to 1.06 ± 0.35 mg/g in September. Ellagic acid content also peaked in May at 3.77 ± 1.01 mg/g, then continuously decreased before increasing again to 1.40 ± 0.71 mg/g in September, showing a pattern similar to that of corilagin. Geraniin and neochlorogenic acid contents were also highest in May, at 6.92 ± 3.11 mg/g and 0.45 ± 0.23 mg/g, respectively, and subsequently decreased throughout the cultivation period. Neochlorogenic acid decreased to trace levels after September and could not be reliably quantified thereafter. Corilagin and geraniin are ellagitannin-type polyphenols reported to be associated with the shikimate biosynthesis pathway [36], whereas ellagic acid is a degradation product of ellagitannins [37]. In contrast, neochlorogenic acid is associated with the phenylpropanoid biosynthesis pathway, and differences in its biosynthetic origin may contribute to distinct accumulation patterns [38], as was found in the present study.

**Figure 8:**
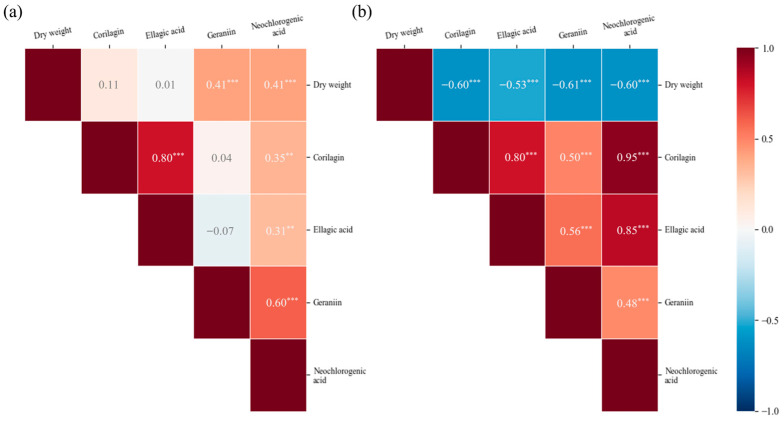
Correlation analysis of (**a**) leaves and (**b**) stems of *P. chinensis* based on dry weight, corilagin, ellagic acid, geraniin, and neochlorogenic acid. Asterisks indicate significance levels (*p* < 0.01: **, *p* < 0.001: ***).

Correlation analysis was conducted to evaluate the relationships between dry weight and the contents of corilagin, ellagic acid, geraniin, and neochlorogenic acid in the leaves and stems of *P. chinensis* and to assess whether changes in biomass were associated with proportional changes in polyphenol accumulation (Figure 8). In the leaves, most polyphenol contents showed weak positive correlations with leaf biomass, and weak correlations were also observed among the polyphenols themselves. In contrast, strong positive correlations were observed among polyphenols in the stems, reflecting similar patterns of temporal variation, whereas negative correlations were found between stem biomass and polyphenol content as stem growth continued.

The weak correlations among polyphenols in the leaves are likely attributable to irregular growth patterns associated with leaf abscission during different growth stages.

Overall, these findings suggest that, for the industrial utilization of *P. chinensis*, further cultivation studies focusing on increasing biomass yield and enhancing functional compound accumulation through optimized fertilization and environmental control are required.

## 3. Materials and Methods

### 3.1. Environmental Data Acquisition

Environmental variables in the greenhouse, including temperature and solar irradiance, were automatically recorded using a HOBO data logger (Onset Computer Corp., Bourne, MA, USA). The data logger was installed at a height of 0.8 m above the ground in the center of the greenhouse, and the measurement interval was set to 30 min. The collected data were downloaded using HOBO ware Pro software 3.7.26. and was subsequently used in the statistical analysis.

### 3.2. Plant Materials and Cultivation

*P. chinensis* plants used in the experiment were obtained from the Korea Sejong National Arboretum (management number SJNA2020-002103-001). Subsequently, 8 cm cuttings of *P. chinensis*, collected from genetically identical mother plants, were prepared and propagated for two weeks through hydroponic cutting in a laboratory at the Chungbuk Forest Biocenter, Chungcheongbuk Province, Republic of Korea, maintained at 22 °C and 50% relative humidity, under LED light conditions of 150 µmol·m^−2^·s^−1^ PPFD with a 16 h photoperiod. The propagated individuals were subsequently transferred to a temperature-controlled greenhouse equipped with side vents that opened automatically when the temperature exceeded 25 °C, and were grown individually in 30 cm diameter pots filled with a horticultural substrate (Bioprug, Parmhannong, Jeonbuk, Republic of Korea) from 15 April to 15 October 2025. The aboveground parts were supported with stakes to prevent contact with the ground and avoid mechanical damage during cultivation. Irrigation was supplied using groundwater and delivered automatically via an 8 L·h^−1^ drip system at a constant rate of 1.5 L per plant per day throughout the cultivation period to ensure uniform growth conditions. To evaluate baseline growth characteristics, no additional fertilizers were applied during cultivation. At each sampling time point, 15 plants were harvested on the 15th day of each month using a destructive sampling method. After harvesting, the samples were separated into leaves and stems, and fresh weights were measured on a per-plant basis. Dry weights were then determined after drying at 40 °C for 48 h.

### 3.3. Analysis of Corilagin, Ellagic Acid, Geraniin, and Neochlorogenic Acid

Dried samples collected monthly were subjected to 24 h maceration extraction in a thermostatic water bath (NB-301L, NBIOTEK, Bucheon, Republic of Korea) based on previously reported optimized conditions [8]. All extractions and subsequent quantitative analyses were performed using dry weight, and polyphenol contents are expressed as mg/g DW. Specifically, corilagin, ellagic acid, and neochlorogenic acid were extracted using 80% ethanol at 75 °C by maceration, while geraniin was extracted using 40% ethanol at 50 °C using the same maceration method. The standard markers were of HPLC grade and were obtained from the Natural Product Institute of Science and Technology (Anseong, Republic of Korea). In both cases, a sample-to-solvent ratio of 1:50 (*w*/*v*) was applied. The resulting extracts were centrifuged at 4000 rpm for 20 min and used for subsequent analyses.

### 3.4. Determination of Phenolic Compounds Content Using Reversed-Phase High-Performance Liquid Chromatography (RP-HPLC)

Standard preparations of corilagin, ellagic acid, geraniin, and neochlorogenic acid (1 mg) were weighed and dissolved in 1 mL of ethanol (HPLC grade) to yield a 1 mg/mL stock solution of each phenolic compound, and these were subsequently serially diluted to produce standard solutions with concentrations of 31.25, 62.5, 125, 250, 500, and 1000 ppm for further analyses.

The method used to analyze the corilagin, ellagic acid, geraniin, and neochlorogenic acid contents was based on the procedure outlined by Choi et al. [10]. Prior to injection into an RP-HPLC system, the extract and standard preparations were filtered through a 0.45-μm membrane filter (PVDF: HENKE-JECT, Tuttlingen, Germany). This system consists of a reversed-phase Hypersil GOLD C18 column (250 × 4.6 mm, particle size 5.0 µm) and a Thermo Ultimate 3000 HPLC system (Thermo Scientific, Waltham, MA, USA), equipped with a solvent pump (LPS-3400SD, Thermo Scientific, Waltham, MA, USA), an autosampler (WPS-3000TSL, Thermo Scientific, Waltham, MA, USA), a column oven (TCC-3000SD, Thermo Scientific, Waltham, MA, USA), and a detector (VWD-3400RS, Thermo Scientific, Waltham, MA, USA). The column temperature was maintained at 45 °C. Injected samples (10 µL) were subjected to gradient elution with a mixture of 0.1% formic acid in water and 0.1% formic acid in acetonitrile, the ratio of these two solvents was applied as follows: 0 to 0.5 min, 97:3; 0.5 to 15 min, linear gradient to 87:13; maintained at 87:13 until 55 min; returned to 97:3 at 55.1 min and held until 60 min, at a flow rate of 1.0 mL/min. The concentrations of corilagin, ellagic acid, geraniin, and neochlorogenic acid were measured at 280 nm. Chromatographic analysis was performed using Chromeleon software 7.3.2. (Thermo Scientific, Waltham, MA, USA).

### 3.5. Statistical Analysis

Data were analyzed using one-way analysis of variance, and the means were compared using Duncan’s multiple range test at a significance level of 0.05, using R version 4.5.1. Statistical analyses to evaluate the correlation between the growth characteristics and polyphenol content were conducted using Python 3.12. Data processing and Pearson’s correlation analyses were performed using the pandas library.

## 4. Conclusions

In this study, the growth characteristics and temporal changes in major polyphenol contents of the rare plant *P. chinensis* were investigated under greenhouse cultivation conditions in the central region of Korea. Leaf fresh and dry weights reached their highest values in June, whereas after August, increased leaf abscission led to a decline in leaf biomass in response to reduced solar irradiance and the progression of reproductive growth. The contents of major polyphenols, including corilagin, ellagic acid, geraniin, and neochlorogenic acid, also peaked in June and subsequently showed an overall decreasing trend. In contrast, stem fresh and dry weights increased continuously throughout the cultivation period; however, polyphenol contents in stems were consistently lower than those in leaves and declined after the early growth stage. Differences in accumulation patterns between leaves and stems were associated with variations in biosynthetic pathways, with ellagitannin-type polyphenols being preferentially enriched in the leaves. Notably, this study is the first to demonstrate that *P. chinensis* can stably accumulate relatively high levels of corilagin, ellagic acid, and geraniin compared with other species within the same genus and family, while also confirming its feasibility for continuous production under greenhouse cultivation. These findings indicate that *P. chinensis* represents a plant resource with high commercial potential as an industrial source of functional polyphenols and provide fundamental information for establishing cultivation strategies, including future studies aimed at productivity enhancement through optimized fertilization management and environmental control.

## Data Availability

Data are contained within the article.
